# Flexible cystoscopy‐guided implantation of ProACT: Surgical technique and long‐term outcomes

**DOI:** 10.1002/bco2.70005

**Published:** 2025-03-03

**Authors:** Dimitri Paillusson, Marie‐Liesse De Guerry, Stéphane De Vergie, Marie‐Aimée Perrouin‐Verbe

**Affiliations:** ^1^ Department of Urology Centre Hospitalier Universitaire de Nantes Nantes France

**Keywords:** adjustable ProACT balloons, minimally invasive treatment, postprostatectomy, stress urinary incontinence

## Abstract

**Objectives:**

We aim to describe the ProACT implantation using flexible cystoscopic guidance and to report long‐term outcomes in these patients.

**Patients and Methods:**

This single‐centre retrospective study include all men who underwent ProACT™ for SUI after RP using flexible cystoscopic guidance between 2007 and 2021. The implantation was performed via a perineal approach under general or locoregional anaesthesia. Accurate positioning was ensured using both real‐time fluoroscopic and endoscopic guidance (flexible cystoscopy retroflexed toward the bladder neck).

**Results:**

In total, 196 men were included; 18% (*n* = 36) had previously undergone radiotherapy and 24% (*n* = 46) had undergone SUI surgery. The median (IQR) follow‐up time was 63 (24–108) months. At the last follow‐up, 64% of participants still had their balloon in place, and the success and improvement rates were 62% and 17%, respectively. The perioperative complication rate was 5% (mainly bladder injury and acute urinary retention). Forty‐two per cent (*n* = 82) experienced at least one complication, mainly device deflation (28%). Definitive explantation occurred in 36% (*n* = 71), with secondary implantation of an artificial urinary sphincter in 96% (*n* = 68).

**Conclusion:**

ProACT® adjustable balloon implantation using flexible cystoscopic guidance appears to be an effective and safe long‐term procedure for men with SUI after RP.

## 1 INTRODUCTION

Stress urinary incontinence (SUI) is a well‐known complication of radical prostatectomy (RP), mainly caused by intrinsic sphincter deficiency.^1^ The reported incidence ranges from 4% to 40% depending on the study.^2^ This range of estimations can be explained by differences between surgeons, surgical techniques, and, in particular, differences in SUI definitions.^3^ This complication represents an important issue both economically and in terms of the individual's quality of life.^4^ After RP, around 6% to 9% of patients require SUI surgery.^5^

The first‐line therapy for post RP SUI is non‐invasive treatments such as pelvic floor muscle training.^6^ If this fails, the next option is surgery. The artificial urinary sphincter (AUS) is the gold‐standard treatment for moderate to severe SUI, with a success rate of 60%, and is recommended in the European Association of Urology (EAU) guidelines.^6^, ^7^ However, new minimally invasive devices are available and can be used as a first surgical intervention. Among them, periurethral balloons (Adjustable Continence Therapy ProACT®) are an effective solution, particularly for non‐severe SUI.^6^

ProACT® implantation was initially described by Hübner et al. in 2005 using rigid cystoscopic guidance.^8^ Other implantation methods exist, including the transrectal ultrasound‐guided technique described by Gregori et al. in 2010.^9^ The use of flexible cystoscopic guidance for ACT® and ProACT® implantation was first described in women by Vayleux et al. in 2010^10^ and in men by Luyckx et al. in 2010.^11^

The aim of this study was to determine the long‐term outcomes and the rate of complications of ProACT® adjustable balloon implantation using flexible cystoscopic guidance.

## 2 PATIENTS AND METHODS

### 2.1 Study population

This single‐centre retrospective study included all men who underwent ProACT® balloon implantation between 1 January 2006 and 31 December 2021. The inclusion criteria were SUI after RP, and first implantation of ProACT® using flexible cystoscopic guidance. Exclusion criteria were a follow‐up of less than 12 months.

### 2.2 Surgical procedure

ProACT® periurethral balloon (Adjustable Continence Therapy, Uromedica, Inc.) implantation involves the positioning of two individual silicone balloons on each side of the bladder neck via a perineal approach. Each balloon has an injection port to allow post‐operative volume adjustments.

**FIGURE 1:**
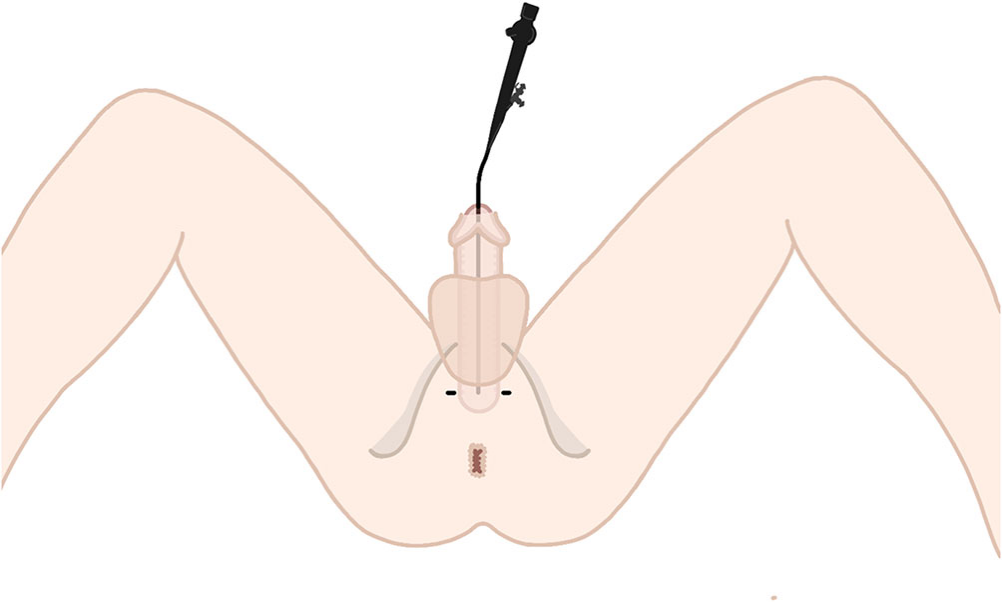
Operator view of perineal incisions.

The procedure is mainly performed on an outpatient basis and either general or locoregional anaesthesia (epidural anaesthesia) is used. The person is positioned in the lithotomy position and a flexible cystoscope is inserted into the bladder and retroflexed toward the bladder neck, allowing simultaneous observation of the bladder neck and bladder filling. Bilateral horizontal perineal incisions (0.5–1 cm) are performed in the perineum to insert the trocars (Figure 1).

**FIGURE 2:**
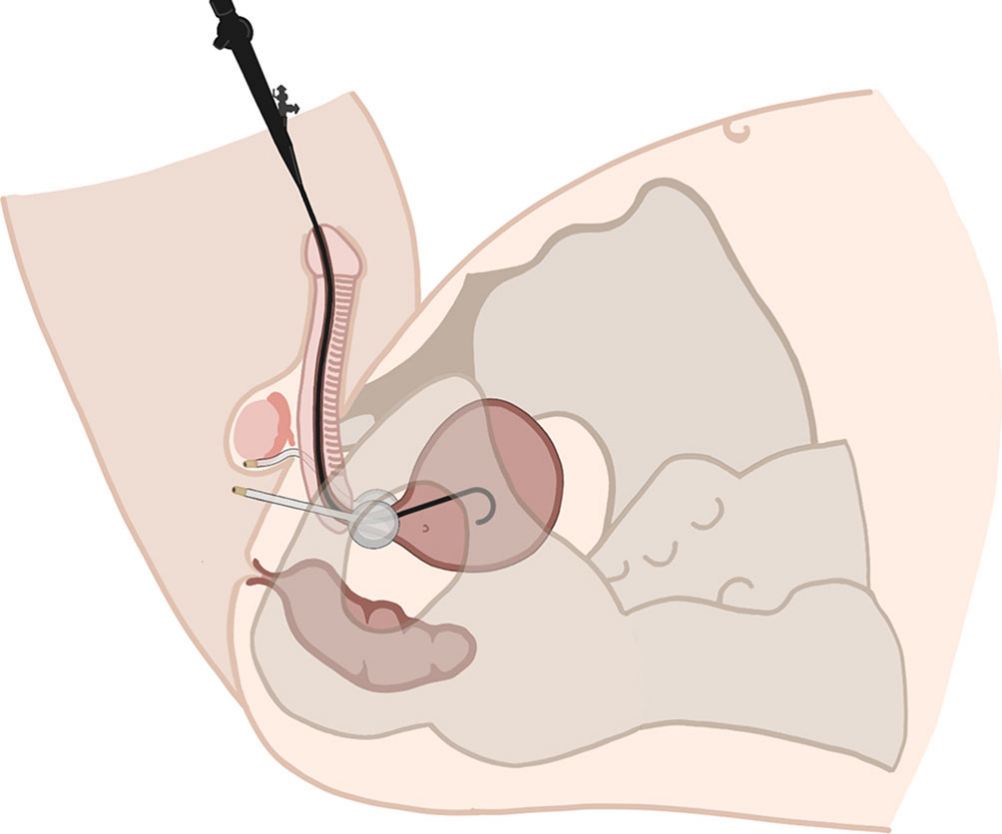
Schematic view of balloon positioning.

A sharp trocar (inserted in the U‐channel sheath) is passed through the endopelvic fascia until it reaches the bladder neck. Both real‐time fluoroscopic and endoscopic guidance are used to accurately position the balloons “en face” in the frontal, transverse and sagittal planes. Ideally, the balloons should be placed at the 3 and 9 o'clock positions to create a triangular coaptation of the urethra between the balloons and the pubis (Figure 2).

Once the ideal position is found, the deflated balloon is inserted in the U‐channel sheath, which is then slightly removed to allow the inflation of the balloon. The balloon is filled with 0.6 mL of an isotonic contrast solution. The same approach is performed on the contralateral side. Finally, titanium ports are implanted subcutaneously under the skin of the scrotum allowing easy access for future percutaneous puncture for adjustments. The incisions are closed with intradermic 4/0 absorbable sutures.

The first adjustment starts at post‐operative week 6 with 0.5 to 1 mL of isotonic contrast solution. Adjustments are repeated every 2 weeks until clinical efficacy is reached.

The surgical procedure is fully described in the video linked to the publication (Data S1).

### 2.3 Data collection

The following preoperative data were collected: age, time since RP, medical history, prior radiotherapy or SUI surgery, and number of pads used per day. Perioperative data were also collected. Patients were then followed post‐operatively for balloons volume adjustments until satisfaction was reached. They were seen at 6 months, 1 year, and then yearly. Additional adjustments were performed if necessary.

At each follow‐up visit, data collected included the Patient Impression of Improvement (PII) measured on a 0–100 numeral rating scale (NRS) reflecting subjective satisfaction, and the number of pads used daily was collected as an objective outcome. If a complication was suspected, a clinical exam was performed, along with an X‐ray and a flexible endoscopy if erosion was suspected.

### 2.4 Endpoints

The primary endpoint was efficacy at the last follow‐up visit. Efficacy was defined using a composite criterion combining subjective (PII) and objective results (daily pad number). Success was defined as ≤1 daily pad associated with an NRS ≥ 80%. Improvement was defined as a decrease of ≥50% in the number of daily pads associated with an NRS ≥ 50%. All other cases were considered as failures.

Efficacy was assessed at 6 months, 1 year, 2 years, and 5 years when available. Perioperative complications were rated using the Clavien–Dindo classification.^12^

### 2.5 Statistical analysis

Continuous variables were expressed as medians (interquartile range [IQR]), and categorical data were expressed as numbers (percentages).

## 3 RESULTS

### 3.1 Population's characteristics

Overall, 212 patients underwent ProACT® implantation using flexible cystoscopic guidance during the study period. Sixteen were excluded: 4 did not have post‐operative follow‐up (two deaths and two lost to follow‐up) and 12 had a follow‐up of less than 1 year. Data from a total of 196 patients were included in the analysis. The median (IQR) follow‐up time was 63 (25–109) months.

Participant characteristics are shown in Table 1. The median age was 68 (64–72) years. The median time between RP and ProACT® implantation was 4 (2–7) years. Thirty‐six patients (18%) had a history of radiotherapy, and 45 (23%) had undergone previous intervention for SUI (AUS or male slings). The severity of the SUI was mild for 101 (51%) patients, moderate for 60 (31%), and severe for 35 (18%, including 21 [11%] with a penile sheath), with a median number of daily pads of two (considering penile sheath as the use of eight daily pads).

**TABLE 1:** Patients' characteristics.

	*N* = 196
Age (years), median (IQR)	68 (64–72)
Follow‐up (months), median (IQR)	63 (24–108)
Time since RP (years), median (IQR)	4 (2–7)
Medical history	
BMI (kg/m^2^), median (IQR)	25 (23–27)
Diabetes, *n* (%)	31 (16%)
Smoking, *n* (%)	73 (37%)
History of radiotherapy, *n* (%)	36 (18%)
History of PFMT, *n* (%)	119 (61%)
History of SUI surgery (male sling), *n* (%)	23 (12%)
History of SUI surgery (AUS), *n* (%)	23 (12%)
Number of daily pads, median (IQR)	2 (1–5)
Severity	
Mild, *n* (%)	101 (51%)
Moderate, *n* (%)	60 (31%)
Severe, *n* (%)	35 (18%)
Including use of penile sheath, *n* (%)	21 (11%)

Abbreviations: AUS, artificial urinary sphincter; PFMT, pelvic floor muscle training; SUI, stress urinary incontinence.

### 3.2 Follow‐up

The number of participants with available data at each endpoint is shown in Table 2.

**TABLE 2:** Success at each follow point in the balloon‐in‐place population (*n* = 125).

	6 months	1 year	2 years	5 years
Success	89 (71.2%)	89 (71.2%)	82 (65.6%)	57 (45.6%)
Failure	36 (28.8%)	36 (28.8%)	20 (16%)	15 (12%)
Lost to follow up	0 (0%)	0 (0%)	23 (18.4%)	53 (42.4%)

The median number of post‐operative visits (including adjustments) was 10. Balloons were filled with a median volume of 5 (3.4–6.6) mL, corresponding to a median of 6 (4–8) post‐operative adjustments.

### 3.3 Efficacy

**FIGURE 3:**
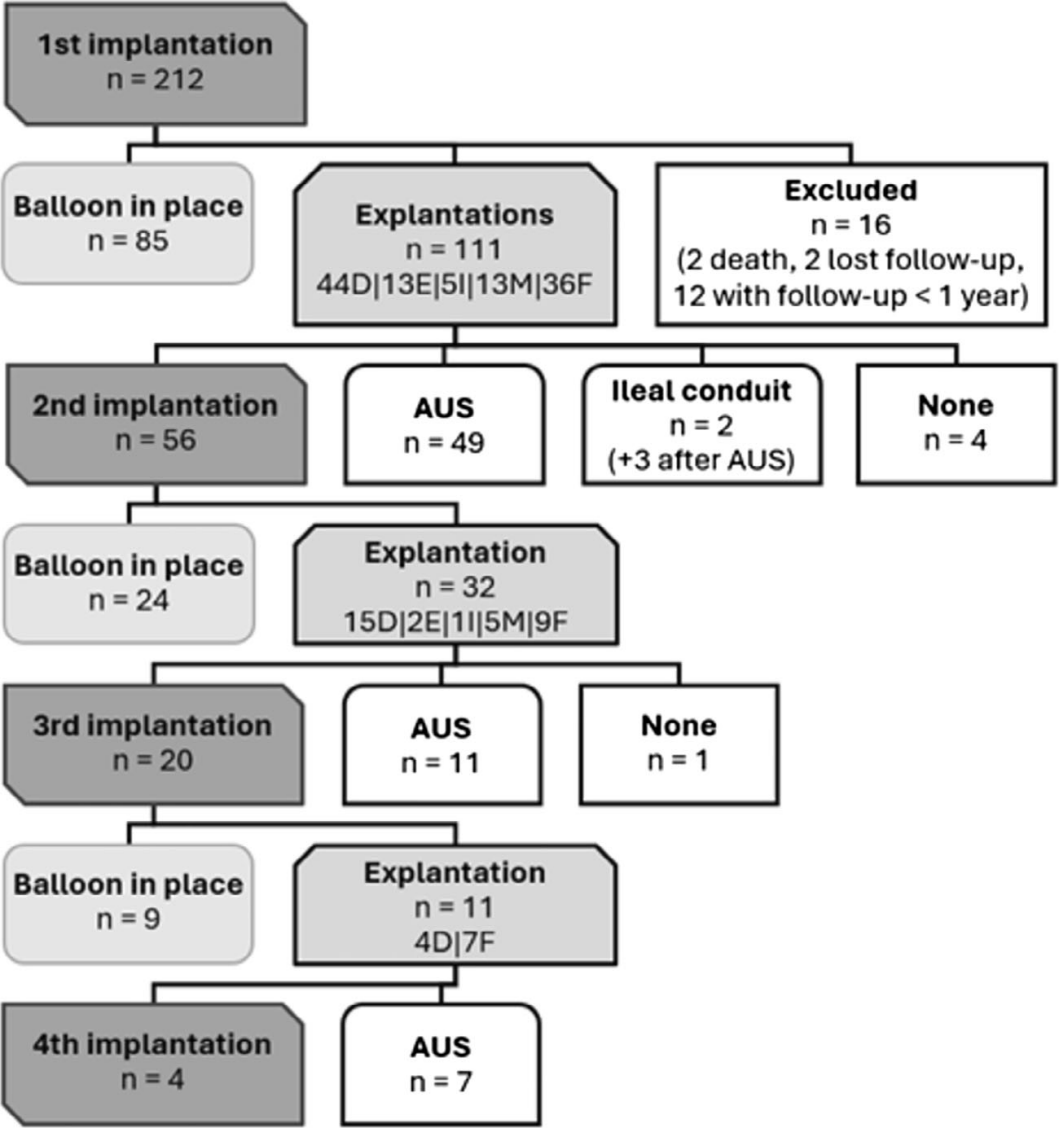
Flowchart (D = deflation, E = erosion, I = infection, M = migration, F = failure).

At the last follow‐up, 125 participants (64%) still had their balloons in place, of which 87 (44%) still had their initial balloon (Figure 3).

In the overall population, at the last follow‐up, the success rate was 41% (*n* = 81), and the improvement rate was 13% (*n* = 25).

The median PII was 70/100 at the last follow‐up, with a median number of daily pads of 1 (versus 2 before balloon placement).

In the balloon‐in‐place group (*n* = 125), success rate was 62%, and improvement rate was 17%. The median PII was 80/100 at the last follow‐up, with a median number of daily pads of 1 (0–3).

**FIGURE 4:**
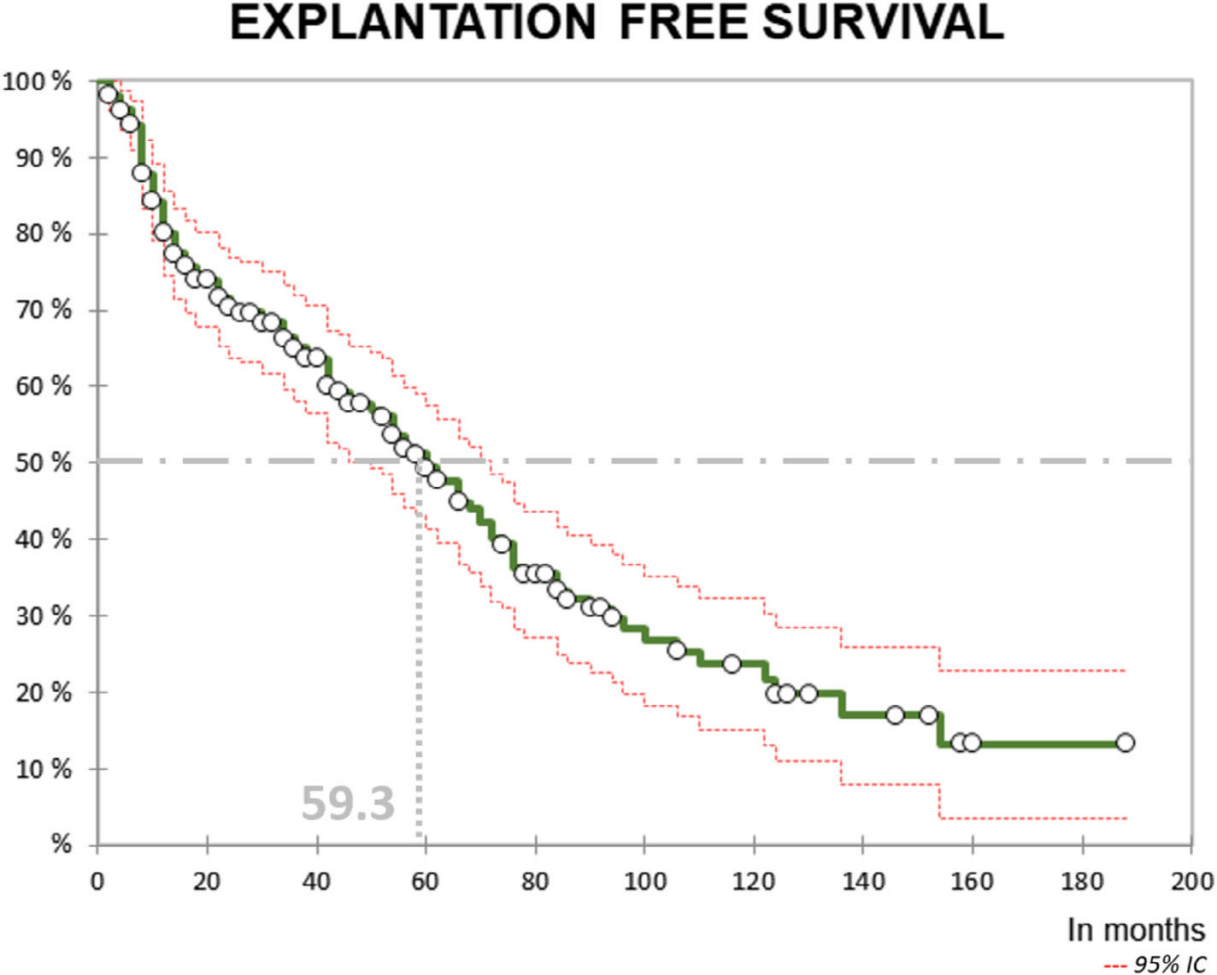
Explantation free survival curve in overall population (first balloon only).

The median explantation free survival was 59.3 months (including only first balloon implanted, Figure 4).

The success rate at each endpoint in the balloon‐in‐place group is shown in Table 2.

### 3.4 Complications

#### 3.4.1 Perioperative complications

Bladder injuries were described in three operative reports, with no further consequences on the procedure (no abandonment of implantation).

#### 3.4.2 Early post‐operative complications

Ten patients experienced early post‐operative complications, eight of which were classified as Clavien I (five cases of acute urinary retention and three hematomas).

Two deaths occurred, with no direct link to the surgery (cardiac failure and acute pulmonary oedema).

#### 3.4.3 Late complications

Throughout the follow‐up, 42% (*n* = 82) of patients experienced at least one complication leading to explantation (definitive explantation or balloon replacement). The most common complications were balloon deflation (*n* = 55, 28%), followed by migration (*n* = 20, 10%), erosion (*n* = 16, 8%), and infection (*n* = 10, 5%).

All these complications were managed by a simple explantation of the device, sometime under local anaesthesia during a consultation.

In total, 28% of patients underwent at least one balloon replacement (including 18% who only underwent one revision).

Seventy‐one patients (36%) underwent definitive balloon explantation, 23 (12%) because of complications and 48 (25%) because of failure. Among them, 68 patients (96%, representing 35% of the whole sample) underwent secondary AUS implantation, 52 (77%) of which were successful.

## 4 DISCUSSION

To our knowledge, this is one of the largest single‐centre series reporting the long‐term outcomes and complications of ProACT® implantation after RP using flexible cystoscopic guidance. This technique has been routinely used in our Department since 2007 and was first described in 2010.^10^, ^11^

This series updates the previously published series by Ricard et al., with four more years of follow‐up, a larger number of patients, and a focus on flexible cystoscopic guidance.^13^ The success rate remains stable (41% in the present study versus 40% previously), but a higher rate of complications has been observed (42% in the present study versus 33% previously), which can be attributed to the longer follow‐up period.

In our opinion, the use of a flexible cystoscopic guidance seems to reduce the learning curve and the risk of bladder injury, especially at the level of the bladder neck if the trocar is inserted too deeply. Indeed, the rate of perioperative bladder injuries in the present series was 2% (*n* = 3), which is lower than that of other series: Hübner et al. reported a 5% injury rate using rigid cystoscopy^8^ and Gregori et al. reported a 3% rate using transrectal ultrasound guidance.^9^ No urethral injuries occurred in our series.

A recent meta‐analysis by Tricard et al (2023), reporting results of 1570 patients from 18 studies, with a median follow‐up of 34.7 months, found 55% success rate, which was higher than that in the present study.^2^ This discrepancy can be attributed to the different definitions of success and continence. When considering continence as 0–1 pad/day in all the articles included in the meta‐analysis, the success rate was 53%. However, this definition does not take into account the subjective improvement of SUI, as we did with the PII in the present study. If we used the same definition of continence, the success rate would increase from 42% to 55% in our series (*n* = 107/196). These results highlight the difficulty of comparing functional results from different studies. Furthermore, the main surgical technique used in the studies included in the meta‐analysis was different from ours (mostly transrectal ultrasound‐guided technique).^9^ In contrast with other large series, our study involved the objective evaluation of a single technique.^14^

A study from 2022 compared rigid and flexible cystoscopy with retrovision of the bladder neck, but failed to find significant differences because of the small size of the subgroup of individuals who underwent rigid cystoscopy.^13^ Another article published in 2010 showed that use of retrovision in women with SUI positively impacted complication and explantation rates, as well as satisfaction scores.^10^

Implantation using flexible cystoscopic guidance could improve balloon placement, providing a greater efficacy with lower volumes. In our series, mean filling was 6 mL in the overall sample, and 4.2 mL in those who experienced improvement or success. Other series found a mean filling of 3.5 mL^8^ to 6.8 mL.^14^ The average number of six adjustments in our series can be explained by the careful injection of 0.5 to 1 mL of saline solution per adjustment to prevent displacement of the device, and to avoid a rapid increase in urethral resistance, which may lead to voiding symptoms.

The reoperation rate of 28% involved at least one device replacement and is consistent with existing literature.

The complication rate of 42% (participants experiencing at least one complication) is higher than that reported in other studies; however, this can primarily be attributed to the longer follow‐up period and, more importantly, to a different definition of complications used in our study as we included all events leading to revision or explantation. Notably, we found a higher rate of device deflation (28% compared to 4% in a meta‐analysis), likely because of the longer follow‐up period.^15^

The explantation rate of 36% is higher than reports in the literature.^2^, ^14^ This higher rate can be also attributed to the longer follow‐up duration, which increases the likelihood of encountering long‐term complications, such as device deflation. However, these results can be mitigated by the relative ease of explantation or replacement of the ProACT® device, compared to AUS.

The results also show that AUS remains an effective solution, with a success rate of 76%, even after failure of previous ProACT® implantation. Other studies have confirmed the feasibility of secondary device implantation after failure of a ProACT® implantation and have shown that this procedure has no impact on potential future surgeries.^2^

Furthermore, due to the different surgical approaches and techniques, ProACT® devices are a viable alternative following AUS explantation or in case of insufficient improvement following a sling procedure.^2^, ^13^, ^16^

## 5 CONCLUSION

Our series of 196 patients followed over 14 years showed good long‐term functional results of ProACT® implantation using flexible cystoscopic guidance. The surgical technique used provided an efficient guidance for this minimally invasive procedure.

## AUTHOR CONTRIBUTIONS

**Dimitri Paillusson:** Conceptualization; methodology; data curation; writing—original draft. **Marie‐Liesse De Guerry:** Investigation; resources. **Stéphane De Vergie:** Resources. **Marie‐Aimée Perrouin‐Verbe:** Conceptualization; methodology; writing—review and editing; supervision.

## CONFLICT OF INTEREST STATEMENT

Dimitri Paillusson, Marie‐Liesse De Guerry and Stéphane De Vergy declare no conflicts of interest. Marie‐Aimée Perrouin‐Verbe proctoring for Uromedica.

## Supporting information


**Data S1.** Supporting Information
